# Supplementary material for: Comparative multiomics analysis of cell physiological state after culture in a basket bioreactor
Source: Sci Rep. 2022 Nov 23;12:20161. doi: 10.1038/s41598-022-24687-4 (PMC9686226; doi:10.1038/s41598-022-24687-4)
Supplement: Supplementary file 1 — Supplementary Information 1. [file 41598_2022_24687_MOESM1_ESM.zip › raw data/Proteomic raw data/6.KEGGenrichment/EG.vs.CG/UP/EG.vs.CG.KEGG_Enrich.scatterplot.pdf]

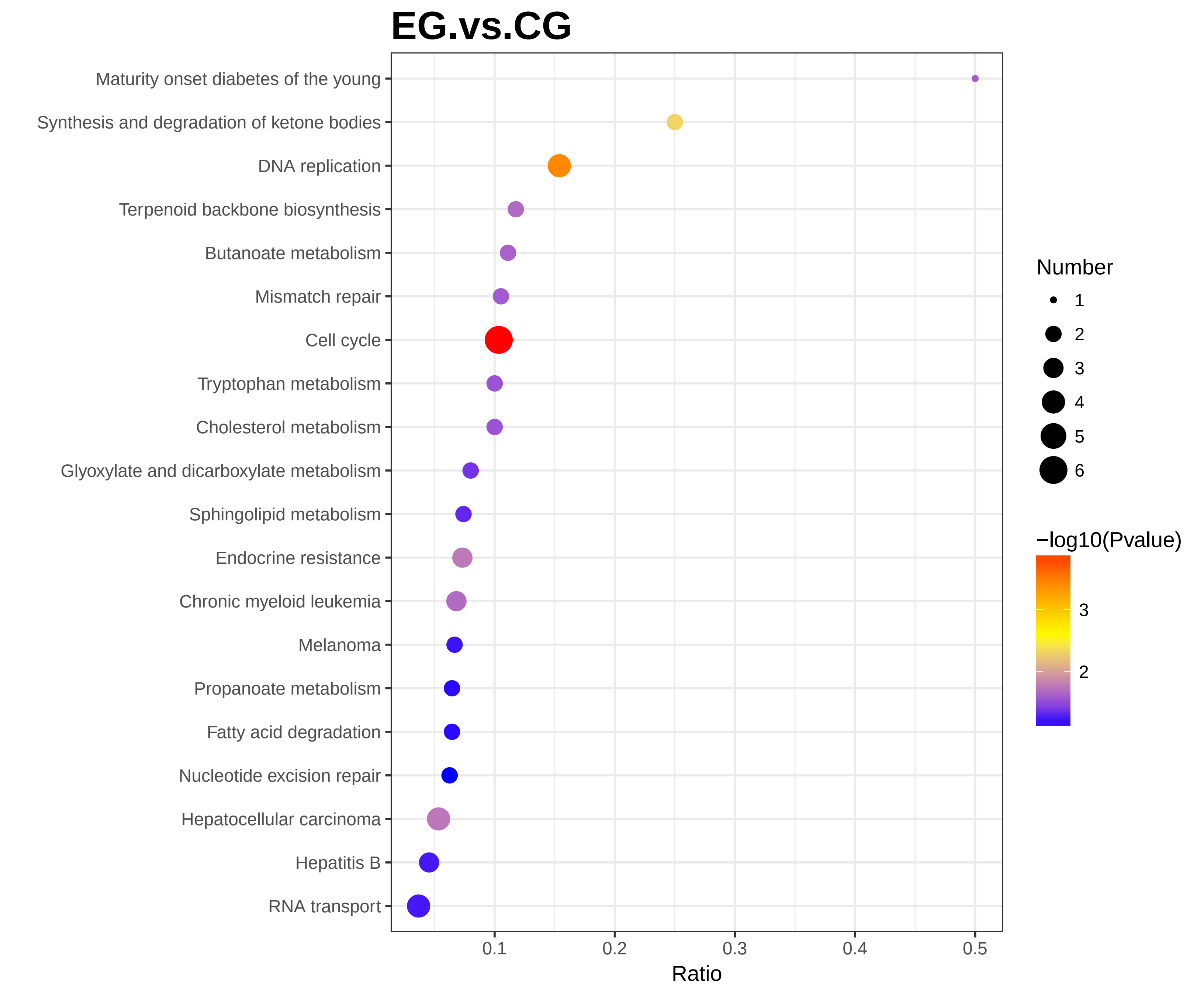
EG.vs.CG
Maturity onset diabetes of the young
Synthesis and degradation of ketone bodies
DNA replication
Terpenoid backbone biosynthesis
Butanoate metabolism
Mismatch repair
Cell cycle
Tryptophan metabolism
Cholesterol metabolism
Glyoxylate and dicarboxylate metabolism
Sphingolipid metabolism
Endocrine resistance
Chronic myeloid leukemia
Melanoma
Propanoate metabolism
Fatty acid degradation
Nucleotide excision repair
Hepatocellular carcinoma
Hepatitis B
RNA transport
0.1
0.2
0.3
0.4
0.5
Ratio
Number
1
2
3
4
5
6
−log10(Pvalue)
3
2
